# The Smartphone in Medicine: A Review of Current and Potential Use Among Physicians and Students

**DOI:** 10.2196/jmir.1994

**Published:** 2012-09-27

**Authors:** Errol Ozdalga, Ark Ozdalga, Neera Ahuja

**Affiliations:** ^1^Stanford University HospitalStanford, CAUnited States; ^2^(not affiliated with an institution)San Jose, CAUnited States

**Keywords:** Smartphone, technology, education, medicine, telemedicine, health care, Android, iPhone, BlackBerry, mobile phone

## Abstract

**Background:**

Advancements in technology have always had major impacts in medicine. The smartphone is one of the most ubiquitous and dynamic trends in communication, in which one’s mobile phone can also be used for communicating via email, performing Internet searches, and using specific applications. The smartphone is one of the fastest growing sectors in the technology industry, and its impact in medicine has already been significant.

**Objective:**

To provide a comprehensive and up-to-date summary of the role of the smartphone in medicine by highlighting the ways in which it can enhance continuing medical education, patient care, and communication. We also examine the evidence base for this technology.

**Methods:**

We conducted a review of all published uses of the smartphone that could be applicable to the field of medicine and medical education with the exclusion of only surgical-related uses.

**Results:**

In the 60 studies that were identified, we found many uses for the smartphone in medicine; however, we also found that very few high-quality studies exist to help us understand how best to use this technology.

**Conclusions:**

While the smartphone’s role in medicine and education appears promising and exciting, more high-quality studies are needed to better understand the role it will have in this field. We recommend popular smartphone applications for physicians that are lacking in evidence and discuss future studies to support their use.

## Introduction

On April 3, 1973 the first cellular phone call was placed by a general manager at Motorola [1]. Ever since, mobile communication has drastically changed the way we work and live our lives. More recently, another technology is again driving such change: the smartphone. Faster processors, improved memory, and smaller batteries in concert with highly efficient operating systems capable of advanced functions have paved the way for applications (commonly referred to as apps) that are affecting our personal and work environments. Like other industries, the field of medicine experienced the resounding effects of the smartphone. In fact, it may be among those industries where the impact has been most profound. One market research firm estimates that 72% of US physicians use a smartphone, and the research firm expects this number to rise to 81% in 2012 [2]. In another study, 85% of medical providers working in Accreditation Council for Graduate Medical Education training programs reported use of the smartphone [3].

 Today, smartphones are being manufactured by numerous companies and are one of the fastest growing sectors in the technology industry. Operating systems include Google’s Android, Apple’s iOS, Research in Motion’s BlackBerry, Nokia’s Symbian, and the Windows Phone 7 platform. From patient monitoring and diagnostics to more efficient medical education and communication, smartphones serve a vital role in the practice of medicine today. In this review, we will the available literature to understand how the smartphone has changed the field of internal medicine and medical education. We also survey the ways in which the smartphone is used to better understand how that impact might be achieved. We conclude this review with suggested apps for physicians based on anecdotal experience and suggest studies that can better answer these questions.

## Methods

### Database Search

We searched both Medline via PubMed and Scopus databases for the literature review. Using PubMed we searched a combination of the Medical Subject Heading *cellular phone *with the related key words smartphone*, smart phone*, *mobile phone*, *iPhone*, *android*, *blackberry*, and *windows mobile*. All terms were combined using the Boolean operator OR. Our Scopus search used the following keywords: smartphone*, smart phone*, *iPhone*, *android*, *blackberry*, and *windows mobile*, and was combined with *medicine *to narrow our results. We limited our search to journals written in English in both databases. Both searches were also limited to the last 5 years. The citations of the search results were then manually queried for eligible articles and reviews. These searches were conducted over a period of 10 months, from August 2011 to May 2012.

### Search Criteria

Given the broad nature of our review, we attempted to include all articles that either exemplified particular roles for the smartphone in health care or discussed its implications. For the purposes of this article, we defined the smartphone as any cellular device that has additional functions including a camera, global positioning system (GPS), and Wi-Fi capabilities and is running one of the following operating systems: iPhone, Android, BlackBerry, or Windows Mobile. Our search criteria included any primary article or review that discussed innovative roles of the smartphone in the field of internal medicine. These roles included patient care, medical reference, and continuing education. We also sought uses of the smartphone in medical education, communication, and research. We excluded any articles that were limited to mobile phones (considered the precursor to the smartphone) or personal digital assistants. Additionally, we did not include papers within the field of surgery or the surgical subspecialties given the already broad scope of this topic. Moreover, as internal medicine physicians, we felt it would be inappropriate to comment on the uses of the smartphone in this setting.

 Research on the use of smartphones in medicine and medical education is limited, which hindered our development of a systematic article selection process. We did give priority to primary sources that were controlled, multicentered reports providing outcome data.

## Results

### Search Results

**Figure 1:**
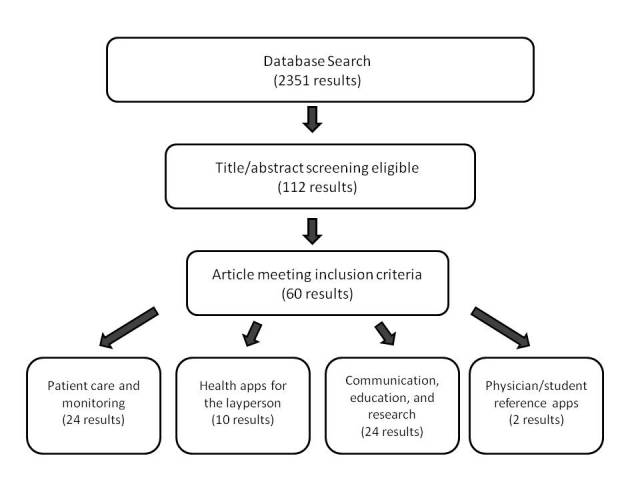
Flow diagram of the process of article selection and results.

From our initial combined database search, we retrieved 2351 articles (Figure 1). A title and abstract review was conducted, from which we identified 112 articles for detailed review and added 1 article from the citation review. Of these, 60 articles met the eligibility criteria. Given the large sample size, we further analyzed these articles to subdivide them into the following categories: (1) patient care and monitoring, (2) health apps for the layperson, (3) communication, education, and research, and (4) physician or student reference apps. Of note, some papers involved both patient care and communication, but we categorized them based on the smartphone’s primary purpose. For example, if the smartphone was aiding patient care via telediagnosis, then we placed the article in the category of communication.

### Patient Care and Monitoring

Our literature search found several examples of the use of the smartphone’s features for patient monitoring. One such example involved patients with Alzheimer disease. An attempt to deal with the risk of wandering was proposed with the use of the Android app iWander [4]. The app works by using the smartphone’s GPS to track the patient at all times. The patient’s age, level of dementia, and home location on the GPS are input into the software. If the GPS detects that the patient is away from his or her home (for example, uncharacteristically late in the day or during inclement weather), the algorithm may predict that the patient has become confused. The app then requests that the patient manually confirm his or her status. Not providing confirmation triggers an alarm that notifies the patient’s family and primary care doctor or contacts emergency personnel. It has also been suggested that identifying Alzheimer patients with depression might be possible simply by monitoring behaviors via the smartphone’s functions, such as their movements using Bluetooth technology, their communication patterns, and their level of activity from the GPS. It is important to note that this app is limited by factors such as GPS and Internet reliability. Also, an older patient with mild dementia may have little ability to use such modern devices.

 The smartphone has also been used in rehabilitation [5]. Using smartphones connected via Bluetooth to a single-lead electrocardiograph (ECG) device, patients who were unable to attend traditional hospital-based rehabilitation were monitored in real time through their smartphones while they exercised in their own neighborhoods [6]. This small study followed 6 patients who recently had a coronary event or angioplasty for 116 exercise sessions. Information obtained from the smartphones allowed researchers to track their patients’ heart rates, single-lead rhythms, locations, altitudes, and walking speeds. This information was then used to create custom exercise regimens, leading to improved postintervention 6-minute walk tests. Patients also reported reduced depression and improved quality of life on questionnaires.

 Other studies demonstrated the smartphone’s potential in patient monitoring. Shoes fitted with sensors that communicate with the smartphone were used to follow the activity level of patients who have recently had a stroke [7]. The smartphone’s accelerometer can be used to interpret gait and balance of patients [8-11]. Another study entailed connecting a single-lead ECG to a smartphone to diagnose and follow treatment with sleep apnea [12], providing a possible alternative to costly and labor-intensive polysomnography. One study used smartphones to promote physical activity by asking participants to routinely log their results [13].

 Recognizing the challenges of a growing elderly population, one group worked on the European Union-funded project Enhanced Complete Ambient Assisted Living Experiment [14]. This project intended to build a comprehensive remote monitoring system targeted at older people with chronic diseases. Using sensors attached to garments, continuous information was monitored and collected. Data from this 3-year project (June 2009 to May 2012) intended to show how smartphone technology provides an environment where older people can maintain their independence. At the same time, the researchers hoped to provide a way to minimize health care costs through early detection of acute illnesses and a decreased need for skilled nursing homes.

 Patients with type 1 diabetes are also among those who could benefit from smartphone technology, by using Diabeo [15]. Diabeo is an app that collects information such as self-measured plasma glucose, carbohydrate counts, and planned physical activity prior to making insulin dosing recommendations. Researchers in France conducted a 6-month multicenter study of 180 adult patients with type 1 diabetes with glycated hemoglobin above 8%. They found that patients using Diabeo together with telephone conversations had lower glycated hemoglobin levels than those with clinic visits. The app was used safely with no differences in hypoglycemic events.

 The ability to automatically monitor patients with diabetic and heart conditions from their smartphones is being developed [16]. This technology extends to other conditions such as movement disorders or bipolar disorder [17-19]. Additionally, engineers are testing the smartphone to be used as a device for monitoring patients’ balance using the phone’s accelerometer [20].

 The use of the smartphone as a patient-monitoring device has also been described in resource-poor countries. Smartphones used by health care workers treating malaria in rural Thailand allowed for better follow-up, medication adherence, and collection of information [21]. A similar study in Kenya allowed workers to collect data during home visits [22].

 With a hands-free microphone, the smartphone has been used to record heart sounds for tracking heart rate and heart rate variability. The phone’s camera along with its light-emitting diode light source has been shown to measure heart rate accurately [23]. Recently, teams have begun working on ECG recording devices that work with smartphones [24,25]. Moreover, the smartphone is being used for echocardiography [26]. MobiSante (MobiSante, Inc, Redmond, WA, USA) became the first company to design and build a US Food and Drug Administration (FDA)-approved cell phone-based medical diagnostic tool with an ultrasound probe in January 2011. A smartphone connected to a Doppler device has been used for blood flow measurement [27].

### Health Apps for the Layperson

In our review we did not find clear data describing usage trends of apps for patients. Additionally, we did not find any evidence that these apps lead to wellness. Here we briefly review some apps mentioned in our literature search for laypersons.

 Weight loss and fitness apps are among the most used. The apps Lose It! and Calorie Counter provide a way for people to keep track of how many calories they consume and burn for better control of their weight loss goals [28]. Based on the input information, such as the type and quantity of food consumed, these apps calculate the user’s total daily caloric expenditure.

 Other apps help track the amount of exercise an individual does. Using the GPS and accelerometer, phones can be turned into and navigators and pedometers [28].

 Wellness apps that teach yoga are available, as are apps that focus on other forms of relaxation such as breathing [28]. Women can input the dates of their periods and body temperature to help predict ovulation. Some apps remind a patient to take his or her medication. Other apps contain an individual’s important medical information such as allergies, medications, and contact phone numbers in the event of an emergency. There is also an iPhone app that offers free hearing tests [29].

 Of the available data concerning the validity of apps to promote wellness, a review of obesity-related apps for diet and exercise showed that a vast majority of them rated low on a custom scoring system based on topics covered, accuracy, and other parameters [30]. Similar findings were noted with reviews of apps for alcohol abuse and smoking cessation [31,32].

 One report described a method of surveying participants to create a framework from which to create an app promoting physical activity [33].

 iTriage (iTriage, LLC, Denver, CO, USA) is an app that provides patients with information such as the locations of nearby emergency rooms, doctors by specialty, and other practical information [34]. It provides emergency room wait times and allows for registration via the app at participating locations. Another similar app was designed to improve diagnosis and treatment times of stroke patients [35]. ZocDoc (ZocDoc, Inc, New York, NY, USA) allows patients to conveniently make appointments with physicians who choose to use this system. Patients can view open slots and other information about participating doctors.

### Communication, Education, and Research

The smartphone has been used for years in hospitals with limited network capability [36]. It also has been shown to improve communication among doctors and nurses on inpatient wards. Timely communication within hospitals remains a fundamental means by which to reduce medical errors [37]. The internal medicine program at Toronto General Hospital conducted a study using dedicated BlackBerrys for each medical team [38]. Nurses could call the team or use a Web program to send emails to these phones for less-urgent issues. Overall, surveys from residents reported improvements in communication and decreased disruption of workflow. Nurses reported decreased time spent attempting to contact physicians; however, there was no change in response time for urgent issues. Another study by this group also illustrated the efficiency of smartphones over pagers but noted a perceived increase in interruptions and weakened interprofessional relationships [39]. They also reported value in the ability to receive nonurgent messages via email; however, there has been disagreement as to what types of messages are appropriate for various communication methods [40].

 Communication is also affected by integrating with electronic medical records. One company, Epic Systems (Epic Systems Corporation, Verona, WI, USA) has partnered with Apple (Apple Inc, Cupertino, CA, USA), releasing versions of Epic for both the iPhone and iPad [41]. Another app specifically designed for one particular hospital is being created to provide doctors with access to patient records from smartphones [42].

 Interesting and educational patient physical findings are better documented with the use of the phone’s camera. A group demonstrated that they could accurately diagnose acute stroke on brain computed tomography scans through the use of iPhones with identical accuracy to standard workstations [43]. Another study of stroke patients found comparable examinations of patients in person and via iPhone [44].

 Several examples demonstrating the smartphone’s role in communication can be found in developing countries with scarce resources [45]. In Africa, the amount of network coverage to send text messages with pictures ranges from 1.5% to 92.2% [46], providing an opportunity to send pictures of physical findings to aid in telediagnosis. Pictures from phone cameras of Gram stains have been sent via text messaged for remote diagnosis [47]. Video clips of limited echocardiographic studies were taken in remote Honduran villages sent via iPhones to experts for interpretation [48]. This has been reproduced with lung ultrasound [49]. Engineers have created various microscopes that attach to smartphones, providing a cost-effective and mobile way to bring more technology to poor and rural regions [50-52]. Development of point-of-care apps for human immunodeficiency virus (HIV) infection treatment to support physicians with limited HIV training in undeveloped regions is expected to minimize errors and improve outcomes [53].

 Outbreaks Near Me (HealthMap, Boston Children’s Hospital, Boston, MA, USA) provides information on disease outbreaks by geography [28,54]. This project, funded by Google and done in collaboration with organizations including the US Centers for Disease Control and Prevention, obtains information from multiple resources, including online news, eyewitness accounts, and official reports.

 In one example of the uses of the smartphone in medical education, doctors who were trained to use a smartphone app for teaching advanced life support had significantly improved scores during cardiac arrest simulation testing [55]. A survey among medical residents in Botswana showed how a smartphone preinstalled with medical apps can be an effective way to obtain information in a resource-poor region [56].

 We found two articles describing the use of the smartphone in medical research. In one example, the smartphone was used to improve data collection during trials [57]. In another, a study of falls risk in the elderly, the smartphone’s accelerometer was used to help detect those at higher risk [58].

### Physician and Student Reference Apps

During our literature review, we found very limited data regarding the use of reference apps by physicians or medical students.

 A study in 2010 claimed that over 60% of physicians surveyed felt that Epocrates (Epocrates, Inc, San Mateo, CA, USA) helped to reduce medical errors [59]. Epocrates claims that their app can help save 20 minutes of time each day for many of their users [60], but this is not supported by the evidence base.

## Discussion

Here we summarize the database search identifying how the smartphone is being used in medicine. We focused on data that would either support or negate the impact of the smartphone and then surveyed the range of uses to better understand the forms in which that impact might occur.

 With respect to patient care and monitoring, we found various ways of using the smartphone to monitor patients. We identified research attempting to provide evidence that the smartphone has advantages in this area; however, much of this is still in the preliminary phase. Apps such as iWander for people with dementia (see Patient Care and Monitoring above) could improve quality of life and decrease financial burden. Approximately 5.3 million Americans have Alzheimer disease, and it has been estimated that this number could quadruple by the year 2050 [61]. As we enter a new era of rising medical costs exacerbated by a growing elderly population, our health care system is looking for ways to meet the rising demand. It remains to be seen whether the smartphone can help.

 We found a wide range of apps for the layperson, from wellness apps to apps that allow improved communication with health care providers. The greatest concern is the general lack of regulation and an evidence base for many of these wellness apps. Much like the general information available on websites, the content of many health-related apps is poorly scrutinized for accuracy. The FDA does not control the content of most apps; only when apps cross the line of providing direct medical advice does the FDA make approval mandatory. An example of this is the diabetes app WellDoc DiabetesManager System (WellDoc, Inc, Baltimore, MD, USA), which required FDA approval when it started providing medical advice based on input blood glucose levels. Other apps available for free download include symptom checkers, where people can input basic symptoms such as abdominal pain and get a whole list of possible causes, prompting inappropriate self-diagnosis and unneeded anxiety.

 Not surprisingly, we found a larger number of articles that discussed ways in which the smartphone is improving communication on internal medicine wards. These results suggest that there may be a role for better communication between doctors and nurses; however, drawbacks such as weakened interprofessional relationships may produce new issues. We found it interesting that many people are looking into using the smartphone for remote diagnosis. It is easy to imagine the huge benefits that could be reaped in resource-poor regions of the world, but this may also lead to a change in insurance companies’ reimbursement methods. In the future, patients may not need to see their physicians in person as often to get the same quality of care. However, we have not found any evidence specific to the smartphone to support this.

 We also find some examples of the smartphone’s use in education, such as a program that teaches cardiopulmonary resuscitation, and the use of smartphones by students in resource-poor countries. Medical schools in the United States are also beginning to use technology more in their curriculum. For example, at Stanford University’s medical school all students are given an iPad to use in place of text books. Online resources are easily accessed.

 As the role of the smartphone continues to grow, we can only expect that its role in medical education will expand with more institutions incorporating it into their curriculums. However, we need to have better evidence to support both its use and methods of how best to use it.

 Among our categories, we found that the area most lacking in evidence is the use of smartphones for physician or student reference apps. We found only one older study looking into the effectiveness of apps to aid in the practice of medicine, with Epocrates. There is no clear reported data on usage statistics, but we presume that given the high number physicians with smartphones and the large availability of apps, many physicians are using these reference apps. A survey of health care providers showed that attitudes toward using smartphones are in general very positive [62].

### Medical Reference Apps

Given the importance of medical reference apps and the paucity of published data regarding available apps and the evidence for their use, we present a list of commonly used apps and make suggestions for future research toward better understanding their utility. This list of apps derives from our anecdotal experience, for which we have given preference to apps known to have a vast database, to have reliable content, to be well respected (or contain information adapted from well-respected resources), and to have been available for many years. There are no conflicts of interest. We review some of the most popular and important apps being used to enhance continuing medical education, improve patient care, and promote communication (Table 1).

**Table 1:** List and description of popular medical applications for physicians.

Smartphone application	Description
Epocrates	Free up-to-date pharmacologic reference and paid medical reference.
DynaMed	Medical and pharmacologic reference
Johns Hopkins’ Antibiotics Guide	Antimicrobial reference
Sanford Guide to Antimicrobial Therapy	Antimicrobial reference
Diagnosaurus	Differential diagnosis
Taber’s Medical Dictionary, Stedman’s Medical Dictionary, and Dorland’s Medical Dictionary	Medical dictionaries and reference
Archimedes (Archimedes 360°)	Free medical calculator (Archimedes 360° is available for a fee)
AHRQ ePSS^a^	Primary care prevention
Medscape	Medical reference, news, and education
Massachusetts General Hospital’s Pocket Medicine	Medical reference
Washington Manual of Medical Therapeutics	Medical reference
QuantiaMD	Medical education with interactive cases
MedPage Today	Medical news
Doximity	Social networking for physicians and physician communication

^a ^Agency for Healthcare Research and Quality Electronic Preventive Services Selector.

 Epocrates is well known for offering a free, up-to-date pharmacologic reference that is available for all smartphone platforms. Strengths of this program include drug dosage guidelines, adverse reactions, mechanism of action, and a drug interaction checker. Epocrates also offers an upgrade to the full version that includes more comprehensive disease and laboratory information. Another resource for pharmacologic reference is mobilePDR (PDR Network, LLC, Montvale, NJ, USA), available for free to doctors after validation of credentials.

 Another resource in medical-related apps for doctors is Skyscape (Skyscape.com, Inc., Marlborough, MA, USA). Skyscape focuses on creating mobile phone apps for all health care professions and boasts over 600 apps spanning 35 specialties. They formulate popular textbooks into searchable programs on the smartphone. There are many notable apps, including Massachusetts General Hospital’s Pocket Medicine and The Washington Manual of Medical Therapeutics. Unbound Medicine (Unbound Medicine, Inc, Charlottesville, VA, USA) produces a similar product offering. Both companies have apps available for many categories, including pharmacology, medical references, and medical dictionaries.

**Figure 2:**
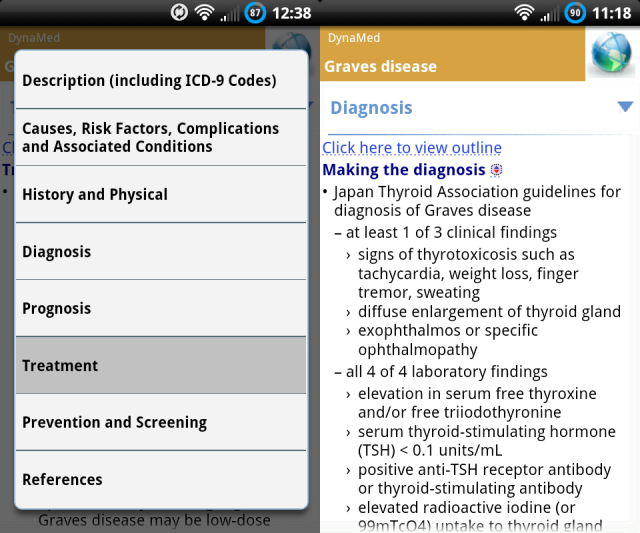
DynaMed’s medical reference program with organization of topics shown at left.

 DynaMed (Figure 2) is a full medical reference app from EBSCO Publishing (EBSCO Publishing Inc, Ipswich, MA, USA). DynaMed houses a large repository of disease, syndrome, and drug information. It differs from other resources such as UpToDate (UpToDate, Inc, Waltham, MA, USA) in that it optimizes its content for use and display on a mobile platform. Once the app is fully downloaded, an Internet connection is no longer needed to access this program. 5-Minute Clinical Consult (Lippincott Williams & Wilkins, Philadelphia, PA, USA) is another medical reference app organized similarly to DynaMed. UpToDate offers a mobile Web version of their ubiquitous Web platform that is similar in organization but does not offer a smartphone app.

 A popular infectious diseases resource is Johns Hopkins’ Antibiotic Guide (Unbound Medicine, Inc), providing detailed information regarding antibiotics and pathogens. Another well-known antibiotic resource is the Sanford Guide to Antimicrobial Therapy (Antimicrobial Therapy, Inc, Sperryville, VA, USA).

 Mobile differential diagnosis programs can help ensure that common diagnoses are not overlooked or discovered too late. One such well-known program is Diagnosaurus (The McGraw-Hill Companies, Inc; New York, NY, USA).

 Other apps, such as medical calculators, are very prevalent in app stores and can help quickly calculate risk scores or other common calculations, such as water deficit in hypernatremia. Skyscape offers a free medical calculator called Archimedes. Lastly, medical dictionaries such as Taber’s, Stedman’s, and Dorland’s are invaluable resources to have readily available in one’s pocket.

 An app for primary prevention is offered for free by the US Department of Health and Human Services, Agency for Healthcare Research and Quality (AHRQ). The AHRQ Electronic Preventive Services Selector is an app designed to assist primary care physicians in identifying screening, counseling, and preventive measures based on their patient’s age, sex, and other risk factors.

**Figure 3:**
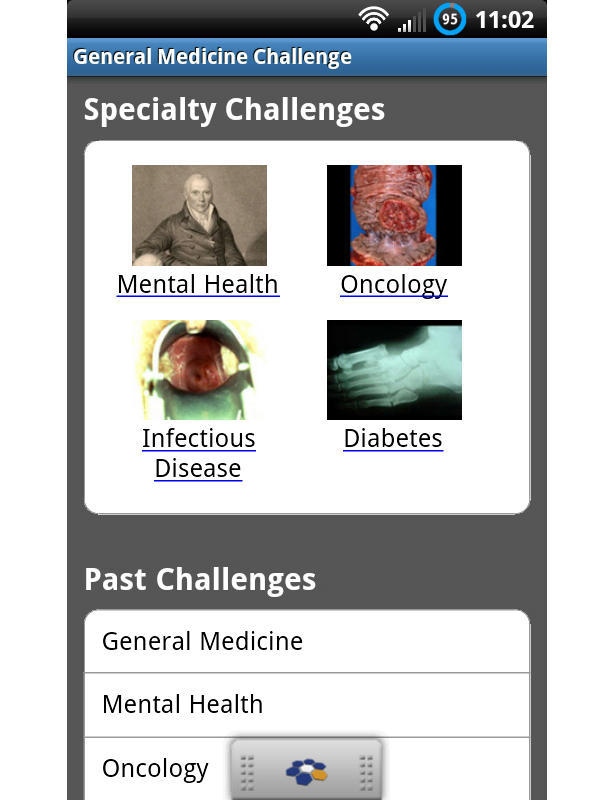
QuantiaMD allows for interactive case challenges within multiple specialties, features guest lecturers, and allows physicians to earn continuing medical education points, all from the smartphone.

 The aforementioned programs are just a few of the large number of evolving resources on the mobile phone. Companies such as Medscape (Medscape, LLC, New York, NY, USA) offer a mobile resource for medical and drug information. They also provide medical news and case studies for continuing education. QxMD (QxMD Software Inc, Vancouver, BC, Canada) specializes in mobile medical programs such as their popular ECG Guide. Information from the *International Classification of Diseases*, 9th revision (ICD-9) can be quickly obtained from QxMD’s ICD-9 app. DynaMed’s app also contains ICD-9 information. QuantiaMD (Quantia Communications, Inc, Waltham, MA, USA) has a mobile app that specializes in continuing medical education by providing well-scripted interactive case studies that can be shared with colleagues (Figure 3). Continuing medical education credits can also be earned. MedPage Today (MedPage Today, LLC, Little Falls, NJ, USA) allows physicians to stay on top of the latest medical news, organize news by interest, and earn continuing medical education credits.

**Figure 4:**
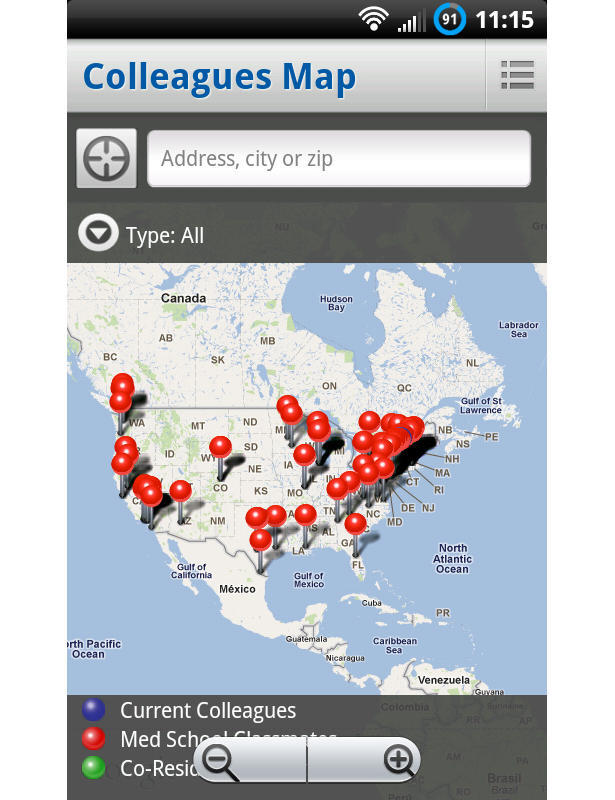
Doximity connects colleagues from around the United States and allows for secure communication.

 Doximity (Doximity Inc, San Mateo, CA, USA) has been likened to a Facebook for doctors. It allows physicians, once registered, to network and even communicate patient-related information in a Health Insurance Portability and Accountability Act-compliant text messaging environment (Figure 4). A credential check of a potential user’s medical license (which is already in their database) is required to sign up.

 While many of these apps have been available for years and are very popular, there are still no data to both support their use and help us understand how best to use them. We believe that studies surveying doctors on the perceived impact that specific apps create, as well as examining patient care outcomes, can help us understand how powerful these apps can be. The use of these apps by students while on clinical rotations can also support education at the bedside. This could translate into an improved quality of education that could be a focus of examination.

### Drawbacks and Obstacles

Although there are numerous benefits to integrating smartphones into the practice of medicine and one’s personal life, there are noteworthy limitations. We again emphasize the ramifications mentioned above of patients self-diagnosing using apps that are not regulated. Moreover, the major technological improvements of both hardware and software are still relatively new and, thus, sometimes unreliable. Furthermore, older physicians and others less inclined to use or intimidated by new technologies may be at a disadvantage if the use of smartphones becomes more requisite within medicine. Similarly, elderly patients may find it difficult to use and interpret the information provided to them by their smartphone, possibly putting them at greater risk than those who are more technologically savvy. And finally, as we become more dependent on technology, we become more dependent on it working flawlessly, with catastrophic implications when it fails.

 Doctors and patients are not able to take full advantage of smartphone technology in areas such as teleconferencing, sending pictures, and emailing, due to health care system reimbursement processes in the United States. These systems usually reimburse only the time spent with patients face-to-face. As the smartphone integrates its way even more permanently into our medical practices, a greater question arises: will this mobile technology improve communication between doctors and patients or detract from it by limiting the personalized interactions that occur best at the bedside or in the office?

### Limitations of This Review

The major limitation of the review stems from the overall paucity of high-quality studies such as multicentered or controlled trials using the smartphone in medicine. While we did find some studies of patient monitoring and communication, even these categories leave many questions to be answered, and future studies are either planned or underway.

 Additionally, we again note that this review did not include papers that demonstrated novel uses of the smartphone in the field of surgery and its surgical subspecialties. As internal medicine physicians, we felt that our analysis on this subject may be inaccurate and thus chose not to include this.

 Another limitation of this study is the rapid and evolving nature of this technology. We intended to make this review as up-to-date as possible, including the addition of new reports just prior to publication of this paper; however, this topic is evolving as rapidly as advancements in the industry are made, outpacing our ability to provide the most current study possible.

### Conclusion

The amount of research in the use of the smartphone in medicine is rapidly growing, but there are very few good-quality studies to answer many questions about its use and the impact it may have. Apps for pharmacology, medical references, and a myriad of other categories are providing physicians with quick and practical medical information that will aid in education and patient care. Communication within hospitals and between patients is improving. Additionally, developing countries now have the potential to access better diagnostic tools in resource-poor regions. However, many obstacles still stand in the way of this progress. The question regarding whether smartphones in medicine will lead to a healthier population with better patient–doctor relationships remains to be answered. Nevertheless, the smartphone has a very bright future in the world of medicine, while doctors, engineers, and others alike continue to contribute more ingenuity to this dynamic field. It is our hope that by informing the medical community of the numerous ways in which the smartphone can be used to benefit health care providers, patients, and their families, the smartphone may one day be recognized as a diagnostic and therapeutic tool that is as irreplaceable as the stethoscope has been in the practice of medicine.

## Abbreviations

AHRQ: Agency for Healthcare Research and Quality
ECG: electrocardiograph
FDA: Food and Drug Administration
GPS: global positioning system
ICD-9: International Classification of Diseases, 9th revision

## References

[1] Lippi G, Plebani M (2011). Laboratory applications for smartphones: risk or opportunity?. Clin Biochem.

[2] Kiser K (2011). 25 ways to use your smartphone. Physicians share their favorite uses and apps. Minn Med.

[3] Franko OI, Tirrell TF (2012). Smartphone App Use Among Medical Providers in ACGME Training Programs. J Med Syst.

[4] Sposaro F, Danielson J, Tyson G (2010). iWander: An Android application for dementia patients. Conf Proc IEEE Eng Med Biol Soc.

[5] Wu HH, Lemaire ED, Baddour N (2011). Change-of-state determination to recognize mobility activities using a BlackBerry smartphone. Conf Proc IEEE Eng Med Biol Soc.

[6] Worringham C, Rojek A, Stewart I (2011). Development and feasibility of a smartphone, ECG and GPS based system for remotely monitoring exercise in cardiac rehabilitation. PLoS One.

[7] Edgar S, Swyka T, Fulk G, Sazonov ES (2010). Wearable shoe-based device for rehabilitation of stroke patients. Conf Proc IEEE Eng Med Biol Soc.

[8] Yamada M, Aoyama T, Mori S, Nishiguchi S, Okamoto K, Ito T, Muto S, Ishihara T, Yoshitomi H, Ito H (2011). Objective assessment of abnormal gait in patients with rheumatoid arthritis using a smartphone. Rheumatol Int.

[9] Nishiguchi S, Yamada M, Nagai K, Mori S, Kajiwara Y, Sonoda T, Yoshimura K, Yoshitomi H, Ito H, Okamoto K, Ito T, Muto S, Ishihara T, Aoyama T (2012). Reliability and validity of gait analysis by android-based smartphone. Telemed J E Health.

[10] Mellone S, Tacconi C, Chiari L (2012). Validity of a Smartphone-based instrumented Timed Up and Go. Gait Posture.

[11] Lee BC, Kim J, Chen S, Sienko KH (2012). Cell phone based balance trainer. J Neuroeng Rehabil.

[12] Bsoul M, Minn H, Tamil L (2011). Apnea MedAssist: real-time sleep apnea monitor using single-lead ECG. IEEE Trans Inf Technol Biomed.

[13] Kirwan M, Duncan MJ, Vandelanotte C, Mummery WK (2012). Using smartphone technology to monitor physical activity in the 10,000 Steps program: a matched case-control trial. J Med Internet Res.

[14] Boulos MN, Wheeler S, Tavares C, Jones R (2011). How smartphones are changing the face of mobile and participatory healthcare: an overview, with example from eCAALYX. Biomed Eng Online.

[15] Charpentier G, Benhamou PY, Dardari D, Clergeot A, Franc S, Schaepelynck-Belicar P, Catargi B, Melki V, Chaillous L, Farret A, Bosson JL, Penfornis A, TeleDiab Study Group (2011). The Diabeo software enabling individualized insulin dose adjustments combined with telemedicine support improves HbA1c in poorly controlled type 1 diabetic patients: a 6-month, randomized, open-label, parallel-group, multicenter trial (TeleDiab 1 Study). Diabetes Care.

[16] Harvey P, Woodward B, Datta S, Mulvaney D (2011). Data acquisition in a wireless diabetic and cardiac monitoring system. Conf Proc IEEE Eng Med Biol Soc.

[17] Kostikis N, Hristu-Varsakelis D, Arnaoutoglou M, Kotsavasiloglou C, Baloyiannis S (2011). Towards remote evaluation of movement disorders via smartphones. Conf Proc IEEE Eng Med Biol Soc.

[18] Puiatti A, Mudda S, Giordano S, Mayora O (2011). Smartphone-centred wearable sensors network for monitoring patients with bipolar disorder. Conf Proc IEEE Eng Med Biol Soc.

[19] Palmerini L, Mellone S, Rocchi L, Chiari L (2011). Dimensionality reduction for the quantitative evaluation of a smartphone-based Timed Up and Go test. Conf Proc IEEE Eng Med Biol Soc.

[20] Rigoberto MM, Toshiyo T, Masaki S (2010). Smart phone as a tool for measuring anticipatory postural adjustments in healthy subjects, a step toward more personalized healthcare. Conf Proc IEEE Eng Med Biol Soc.

[21] Meankaew P, Kaewkungwal J, Khamsiriwatchara A, Khunthong P, Singhasivanon P, Satimai W (2010). Application of mobile-technology for disease and treatment monitoring of malaria in the "Better Border Healthcare Programme". Malar J.

[22] Rajput ZA, Mbugua S, Amadi D, Chepngeno V, Saleem JJ, Anokwa Y, Hartung C, Borriello G, Mamlin BW, Ndege SK, Were MC (2012). Evaluation of an Android-based mHealth system for population surveillance in developing countries. J Am Med Inform Assoc.

[23] Gregoski MJ, Mueller M, Vertegel A, Shaporev A, Jackson BB, Frenzel RM, Sprehn SM, Treiber FA (2012). Development and validation of a smartphone heart rate acquisition application for health promotion and wellness telehealth applications. Int J Telemed Appl.

[24] Hii PC, Chung WY (2011). A comprehensive ubiquitous healthcare solution on an Android™ mobile device. Sensors (Basel).

[25] Oresko JJ, Duschl H, Cheng AC (2010). A wearable smartphone-based platform for real-time cardiovascular disease detection via electrocardiogram processing. IEEE Trans Inf Technol Biomed.

[26] Sicari R, Galderisi M, Voigt JU, Habib G, Zamorano JL, Lancellotti P, Badano LP (2011). The use of pocket-size imaging devices: a position statement of the European Association of Echocardiography. Eur J Echocardiogr.

[27] Huang CC, Lee PY, Chen PY, Liu TY (2012). Design and implementation of a smartphone-based portable ultrasound pulsed-wave Doppler device for blood flow measurement. IEEE Trans Ultrason Ferroelectr Freq Control.

[28] Anonymous (2010). How to put your smartphone "on call." Applications that run on your cell phone put health and wellness aids just a touch away. Harv Womens Health Watch.

[29] Ly K (2011). MHealth: better health through your smartphone. Community Pract.

[30] Gan KO, Allman-Farinelli M (2011). A scientific audit of smartphone applications for the management of obesity. Aust N Z J Public Health.

[31] Cohn AM, Hunter-Reel D, Hagman BT, Mitchell J (2011). Promoting behavior change from alcohol use through mobile technology: the future of ecological momentary assessment. Alcohol Clin Exp Res.

[32] Abroms LC, Padmanabhan N, Thaweethai L, Phillips T (2011). iPhone apps for smoking cessation: a content analysis. Am J Prev Med.

[33] Rabin C, Bock B (2011). Desired features of smartphone applications promoting physical activity. Telemed J E Health.

[34] Anonymous (2011). Smartphone app speeds registration. ED Manag.

[35] Takao H, Murayama Y, Ishibashi T, Karagiozov KL, Abe T (2012). A new support system using a mobile device (smartphone) for diagnostic image display and treatment of stroke. Stroke.

[36] León SA, Fontelo P, Green L, Ackerman M, Liu F (2007). Evidence-based medicine among internal medicine residents in a community hospital program using smart phones. BMC Med Inform Decis Mak.

[37] Wong BM, Quan S, Cheung CM, Morra D, Rossos PG, Sivjee K, Wu R, Etchells EE (2009). Frequency and clinical importance of pages sent to the wrong physician. Arch Intern Med.

[38] Wu RC, Morra D, Quan S, Lai S, Zanjani S, Abrams H, Rossos PG (2010). The use of smartphones for clinical communication on internal medicine wards. J Hosp Med.

[39] Wu R, Rossos P, Quan S, Reeves S, Lo V, Wong B, Cheung M, Morra D (2011). An evaluation of the use of smartphones to communicate between clinicians: a mixed-methods study. J Med Internet Res.

[40] Lo V, Wu RC, Morra D, Lee L, Reeves S (2012). The use of smartphones in general and internal medicine units: a boon or a bane to the promotion of interprofessional collaboration?. J Interprof Care.

[41] Menon A (2011). Confessions of a Wilderness Fellow: I Can't Live Without My Smartphone, Can You?. Perm J.

[42] Choi JS, Yi B, Park JH, Choi K, Jung J, Park SW, Rhee PL (2011). The uses of the smartphone for doctors: an empirical study from samsung medical center. Healthc Inform Res.

[43] Mitchell JR, Sharma P, Modi J, Simpson M, Thomas M, Hill MD, Goyal M (2011). A smartphone client-server teleradiology system for primary diagnosis of acute stroke. J Med Internet Res.

[44] Rahme RJ, Fishman AJ, Hunt Batjer H, Bendok BR (2012). The future is now: smartphones to join scalpels and stethoscopes?. Neurosurgery.

[45] Blaya JA, Fraser HS, Holt B (2010). E-health technologies show promise in developing countries. Health Aff (Millwood).

[46] Bellina L, Missoni E (2009). Mobile cell-phones (M-phones) in telemicroscopy: increasing connectivity of isolated laboratories. Diagn Pathol.

[47] Tice AD (2011). Gram stains and smartphones. Clin Infect Dis.

[48] Choi BG, Mukherjee M, Dala P, Young HA, Tracy CM, Katz RJ, Lewis JF (2011). Interpretation of remotely downloaded pocket-size cardiac ultrasound images on a web-enabled smartphone: validation against workstation evaluation. J Am Soc Echocardiogr.

[49] Crawford I, McBeth PB, Mitchelson M, Tiruta C, Ferguson J, Kirkpatrick AW (2011). Telementorable "just-in-time" lung ultrasound on an iPhone. J Emerg Trauma Shock.

[50] Tseng D, Mudanyali O, Oztoprak C, Isikman SO, Sencan I, Yaglidere O, Ozcan A (2010). Lensfree microscopy on a cellphone. Lab Chip.

[51] Breslauer DN, Maamari RN, Switz NA, Lam WA, Fletcher DA (2009). Mobile phone based clinical microscopy for global health applications. PLoS One.

[52] Zhu H, Yaglidere O, Su TW, Tseng D, Ozcan A (2011). Cost-effective and compact wide-field fluorescent imaging on a cell-phone. Lab Chip.

[53] Sadasivam RS, Gathibandhe V, Tanik MM, Willig JH (2012). Development of a point-of-care HIV/AIDS medication dosing support system using the Android mobile platform. J Med Syst.

[54] Oehler RL, Smith K, Toney JF (2010). Infectious diseases resources for the iPhone. Clin Infect Dis.

[55] Low D, Clark N, Soar J, Padkin A, Stoneham A, Perkins GD, Nolan J (2011). A randomised control trial to determine if use of the iResus© application on a smart phone improves the performance of an advanced life support provider in a simulated medical emergency. Anaesthesia.

[56] Chang AY, Ghose S, Littman-Quinn R, Anolik RB, Kyer A, Mazhani L, Seymour AK, Kovarik CL (2012). Use of mobile learning by resident physicians in Botswana. Telemed J E Health.

[57] Woods CA, Dumbleton K, Jones L, Fonn D (2011). Patient use of smartphones to communicate subjective data in clinical trials. Optom Vis Sci.

[58] Yamada M, Aoyama T, Okamoto K, Nagai K, Tanaka B, Takemura T (2011). Using a Smartphone while walking: a measure of dual-tasking ability as a falls risk assessment tool. Age Ageing.

[59] Rothschild JM, Lee TH, Bae T, Bates DW (2002). Clinician use of a palmtop drug reference guide. J Am Med Inform Assoc.

[60] Terry M (2010). Medical Apps for Smartphones. Telemed J E Health.

[61] Alzheimer's Association (2010). 2010 Alzheimer's disease facts and figures. Alzheimers Dement.

[62] Chen J, Park Y, Putzer GJ (2010). An examination of the components that increase acceptance of smartphones among healthcare professionals. Electron J Health Inform.

